# MYLK Variants of Uncertain Significance as Risk Factors for Aortic Dissection

**DOI:** 10.1016/j.jaccas.2026.109444

**Published:** 2026-07-23

**Authors:** Chanseo Lee, Sedem Dankwa, Michela Cupo, Jaihyoung Lee, Yona Lei, Sriharsha Talapaneni, Irbaz Hameed, Prashanth Vallabhajosyula

**Affiliations:** Division of Cardiac Surgery, Yale School of Medicine, New Haven, Connecticut, USA

**Keywords:** aorta, dissection, genetic disorders, genetics

## Abstract

**Background:**

Myosin light chain kinase (MYLK) variants of uncertain significance (VUS) are not considered actionable under current guidelines, yet MYLK encodes a kinase essential for vascular smooth muscle contractility, and pathogenic variants are associated with aortic dissection at modest diameters.

**Case Summary:**

We report 8 patients carrying MYLK variants (7 VUS, 1 pathogenic) who developed aortic dissection requiring surgery. In our institutional registry of 1,048 patients undergoing aortic genetic panel testing, MYLK carriers had higher rates of acute aortic syndromes, namely Type A dissections.

**Discussion:**

Four phenotypic patterns emerged: dissection at aortic diameters of 40 mm (below guideline thresholds); reoperation at a median of 7 vs 24 months in non-MYLK dissection patients; co-occurring ACTA2 VUS suggesting oligogenic risk amplification; and intrapedigree VUS-to-pathogenic reclassification of the same MYLK V1522A variant across 2 timepoints and testing facilities.

**Conclusions:**

The MYLK variant patient journeys support lower surveillance thresholds and cascade genetic screening.

## Visual Summary

**Figure:**
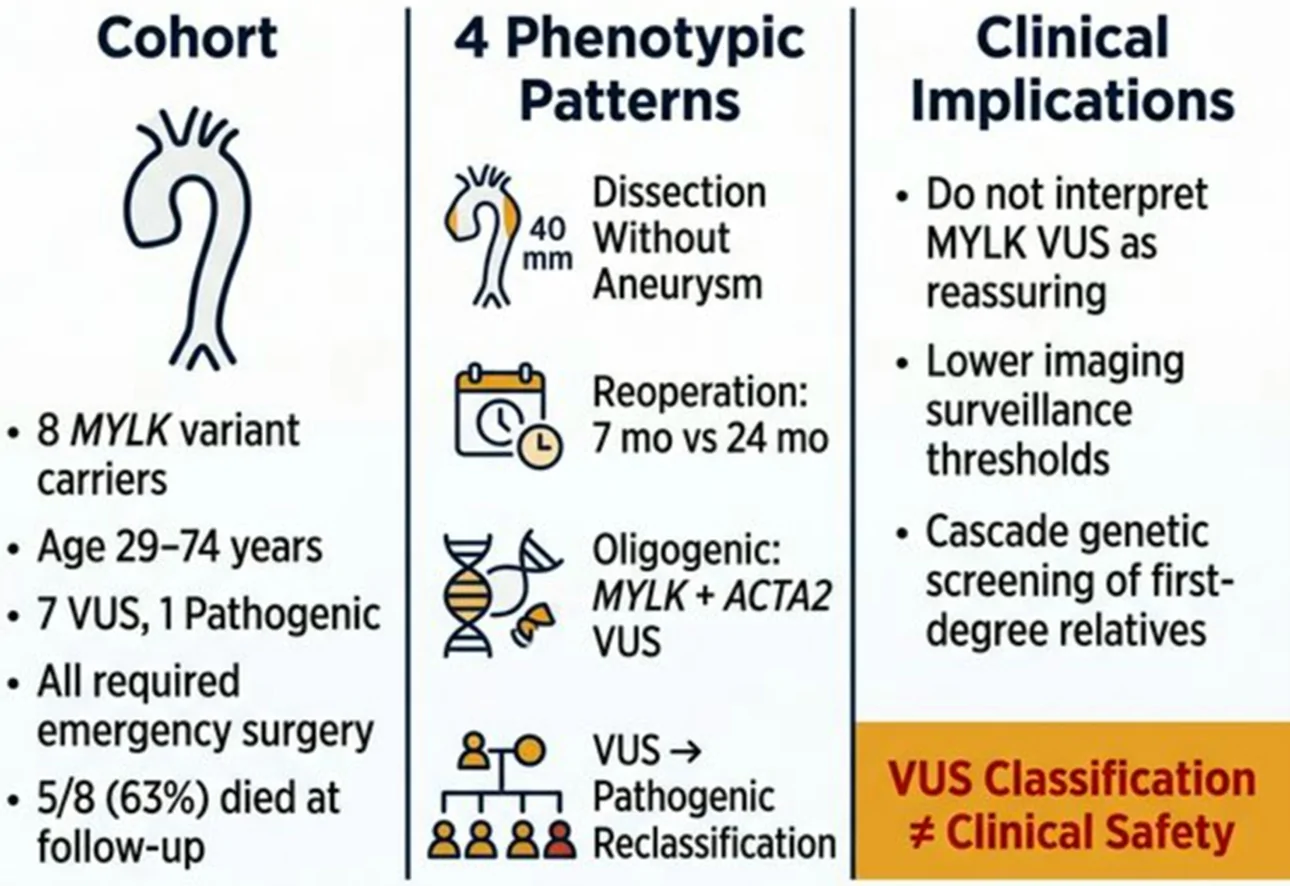

**Visual Summary:**
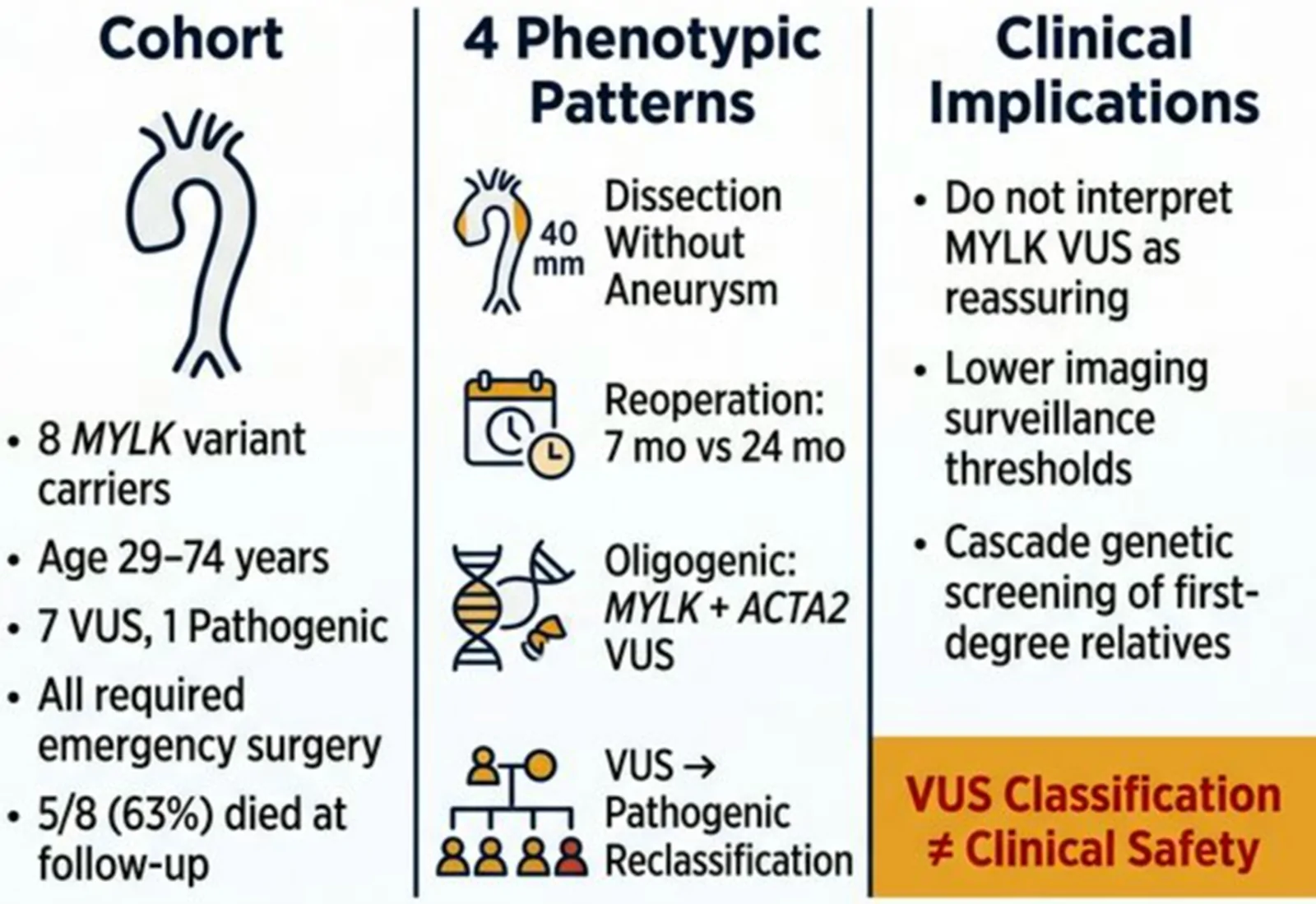
Clinical and Genetic Overview of 8 Patients With MYLK Variants Who Presented With Acute Aortic Dissection We highlight recurrent themes from the case series, including dissection at modest aortic diameters, accelerated postoperative progression requiring reintervention, co-occurring ACTA2 variants suggesting possible oligogenic risk, and familial MYLK variant reclassification supporting cascade genetic screening and longitudinal variant reinterpretation. MYLK = myosin light chain kinase; VUS = variant of uncertain significance.

Thoracic aortic aneurysms (TAAs) often enlarge silently until acute aortic syndromes occur, yielding a narrow intervention window. Acute aortic syndrome incidences are approximately 7.7 per 100,000 person-years, with dissection the most frequent subtype.^1^ Similarly, in the International Registry of Acute Aortic Dissection, mean age was 63 years, classic exam findings were frequently absent, and in-hospital mortality was 27.4%.^2^

**Take-Home Messages**

• MYLK VUS should not be interpreted as clinically reassuring in patients with thoracic aortic disease, particularly when accompanied by dissection at modest diameters, rapid postoperative progression, or a concerning family history.
• These cases support more cautious surveillance, cascade genetic screening, and continued variant reinterpretation as clinical and familial evidence accumulates.

Genetic evaluation has become integral to TAA care because identifying a pathogenic variant can inform earlier gene-based management thresholds.^3^ Major guidelines recommend genetic testing in TAA with syndromic/familial features, as well as screening relatives even with ambiguous or negative genetic testing because of heritable risk.^4^ The same guideline warns that variants of unknown significance (VUSs) are not confirmed to cause TAA, thus cannot inform clinical management.

Myosin light chain kinase (MYLK) encodes a Ca2+/calmodulin-dependent kinase that phosphorylates the regulatory light chain of myosin II to regulate vascular tone.^5^ Pathogenic MYLK variants (often loss-of-function) are associated with nonsyndromic heritable thoracic aortic disease, with acute dissections often occurring with minimal antecedent aortic enlargement. In a study of 33 patients with MYLK, aortic events were predominantly dissections, median age at type A dissection event was only 48 years, and frequently occurred at modest diameters (ascending aorta ranged 3.8-4.8 cm).^6^ These features complicate management and heighten the clinical stakes of uncertain MYLK VUS findings.

We describe 8 patients (Table 1) found to carry MYLK variants who developed acute aortic dissection, contextualized within our institutional TAA registry to inform variant interpretation.

## Case Presentation

### Patient 1

A 29-year-old man with a positive family history of sudden cardiac death in father and uncle presented to the emergency department (ED) due to substernal, left chest pain radiating to neck. He had no previous aortic imaging and no history of connective tissue disease or antihypertensive therapy. Blood pressure 181/104 mm Hg, heart rate 98, temperature 100.1 °F.

Transthoracic echocardiogram (TTE) and computed tomography angiography (CTA) demonstrated a Type A aortic dissection, an aortic root diameter of 55 mm, and preserved left ventricular ejection fraction (60%). Small pericardial effusion and diffuse ST elevation on electrocardiogram were noted with no aortic regurgitation. Genetic testing revealed an MYLK R538W VUS.

The patient was taken to the operating room on the same day. He underwent aortic root replacement, ascending aorta replacement, and transverse hemiarch replacement under deep hypothermic circulatory arrest (DHCA) for 24 minutes. Postoperative intensive care unit course was unremarkable.

The patient was discharged 7 days later on warfarin, regular international normalized ratio checks, and CTA every 6 months. He died 14 months after his index operation for reasons unknown.

### Expert commentary

Patient 1 represents the youngest and most sobering case in this series. At age 29, this patient harbored only an MYLK VUS: a classification that, under current guidelines, does not trigger specific surveillance or lower intervention thresholds. Despite presenting with a 55-mm aorta, the dissection occurred acutely with no previous history. The patient's defining family cardiac history raises the question of whether a strong familial penetrance should prompt aggressive surveillance, genetic testing, and intervention thresholds.

### Patient 2

A 37-year-old male physician and runner presented initially to clinic with pain radiating to the back and lower limbs. Notably, he has hypertension controlled with 5 drugs. He has no recorded family, imaging, or other past medical history.

CTA revealed a descending thoracic aorta (DTA) diameter of 48 mm with substantial narrowing of the lumen, hypothesized to cause his renal hypertension and lower extremity claudication. Genetic evaluation demonstrated 2 VUS: MYLK G1698R and ACTA2 1284N. He was diagnosed with Type B aortic dissection, which was corrected with DTA replacement on left atrial femoral bypass. He subsequently required reoperation 14 months later to replace the remaining proximal DTA because of rapid dilation (5.2 cm on CTA). The patient survived both operations with yearly surveillance on CTA. He remains alive at last follow-up with aortic size not exceeding 3.8 cm.

### Expert commentary

The co-occurrence of MYLK and ACTA2 VUS raises the possibility of oligogenic risk, conferring combinatorial aortic disease severity. Furthermore, this patient required reoperation within 14 months for progressive residual aneurysmal disease, which is far earlier than the mean reoperation interval of 24 months observed in the broader non-MYLK dissection cohort at our institution (n = 27). This may suggest a more rapid progression following initial repair, seen more in this case series.

### Patient 3

A 41-year-old man with a history of ventricular septal defect and aortic coarctation repair on day 3 of life and no follow-up since age 18 presented with acute chest pain radiating to back. He was an active smoker and a heavy alcohol user. He has no family history, no previous imaging, and took no medications.

CTA demonstrated a Type B aortic dissection originating distal to the previous coarctation repair and extending to the celiac artery with a 52-mm DTA diameter. A residual coarctation precluded endovascular repair because of inadequate proximal landing zone. TTE showed left ventricular ejection fraction 55% with mild aortic regurgitation and no pericardial effusion. Cardiology confirmed no residual vs shunt and recommended medical management with anti-impulse therapy. Genetic testing identified an MYLK G139T VUS.

The patient's pain worsened 7 days later, with CTA demonstrating retrograde rupture into the distal aortic arch, contained by adventitia. He was taken emergently for open repair via redo left thoracotomy, requiring adhesions lysis. Replacement of the distal aortic arch (reverse hemiarch) and DTA were performed on cardiopulmonary bypass with DHCA for 26 minutes.

The patient was discharged in 7 days. Seven months later he presented with hypertensive urgency (systolic blood pressure 181 mm Hg) and chest pain; CTA showed stable residual dissection without rupture. He was admitted for blood pressure management and underwent elective thoracic endovascular aortic repair, which was complicated by spinal cord ischemia, and he was discharged 14 days postoperatively. He died 48 months later for reasons unknown.

### Patient 4

A 50-year-old man with a positive family history of TAA in mother and a history of previous percutaneous coronary intervention on dual-antiplatelet therapy presented to outside ED with acute chest pain and a cold, transiently plegic left lower extremity. Echocardiogram 44 months before presentation measured the ascending aorta at 40 mm.

CTA confirmed a Type A dissection involving the ascending aorta and hemiarch, but exact dimensions are not available. An abrupt cutoff of the left common iliac artery was identified, consistent with left leg malperfusion. The intimal tear was located just above the sinotubular junction. Genetic testing revealed an MYLK V1522A VUS.

The patient received ascending aorta and hemiarch replacement via median sternotomy with right femoral artery cannulation (DHCA 15 minutes). Postoperatively, he developed combined cardiogenic and vasodilatory shock requiring vasopressors. He was extubated on postoperative day (POD)1 but became hypoxemic and required reintubation on POD2 with high positive end-expiratory pressure. He developed an acute kidney injury as the left renal artery arose from the false lumen, creating ongoing renal malperfusion, which required aggressive diuresis and renal monitoring. The left lower extremity demonstrated improving Doppler signals. Postoperative intensive care unit course lasted 42 days.

The patient suffered a stroke 50 months postoperation. Surveillance CTA showed a chronic dissection extending from the arch to the right common iliac artery. This appearance was stable since surveillance magnetic resonance angiography 12 months postoperation. Imaging also demonstrated cardiomegaly, bilateral pleural effusions, and anasarca, favoring a chronic volume overload picture.

### Patient 5

A 59-year-old man with a history of hypertension, smoking, hyperlipidemia, obesity, and chronic obstructive pulmonary disease presented to an outside ED with severe back pain and right lower extremity numbness and tingling. His history of sciatica and lumbar disc herniation prompted initial lumbar spine imaging, which identified a suspicious aortic mass. The patient was in hemodynamic shock (blood pressure 85 mm Hg) at the outside hospital, requiring 3 L of fluid and inotropic support before transfer.

CTA confirmed a Type B dissection extending from the aortic valve to the bilateral iliac arteries. The celiac artery, superior mesenteric artery, and inferior mesenteric artery arose from the true lumen; the bilateral renal arteries and innominate artery were dissected. Bedside echocardiogram showed no pericardial effusion. The ascending aorta measured 40 mm. On genetic testing, he carried an MYLK C1339F and ACTA2 intron 2 VUS.

The patient received ascending aorta replacement, deep hemiarch replacement (DHCA 44 minutes), and left femoral artery cannulation. Intraoperative findings included a linearly distributed intimal tear (not the usual transverse distribution), requiring deep hemiarch excision.

Postoperative course was notable for hypertension requiring nicardipine infusion and later oral hydralazine and labetalol. One unit of packed red blood cells was transfused on POD3 for anemia. He was discharged home POD5 on labetalol. The patient died approximately 18 months after surgery from decompensated congestive heart failure.

### Expert commentary

Together, patients 4 and 5 show a concerning motif of dissection at relatively low diameters where no current guideline recommends surgical intervention. It demonstrates a pattern of dissection without significant aneurysm, which has previously been described in ACTA2 pathogenic variants but not in MYLK VUS.^7^ Like patient 2, patient 5 may demonstrate oligogenic amplification of risk.

### Patient 6

A 61-year-old woman with past medical history including hypertension, smoking, chronic obstructive pulmonary disease, HIV, and hepatitis C presented to an outside ED with sudden-onset right-sided chest pain radiating to the right arm, right arm numbness, and altered mentation, vital signs blood pressure 82/46 mm Hg, heart rate 43 beats/min, temperature 95 °F.

TTE identified an aortic dissection flap, and CTA demonstrated a Type A dissection extending from the aortic root to just below the renal arteries, with an ascending aorta diameter of 44 mm. An intramural hematoma of the brachiocephalic artery extended into the right common carotid artery with near-complete occlusion. A left renal artery arising from the false lumen showed global left kidney hypoperfusion and infarction. A computed tomography scan of the chest 11 years previously documented a normal-caliber ascending aorta. Genetic testing revealed an MYLK K241E VUS as the sole identified variant.

The patient was transferred for open repair with ascending aorta and transverse arch (hemiarch) replacement, aortic root repair, and right axillary artery cannulation (DHCA 25 minutes). The patient had an unremarkable postoperative course.

She underwent elective thoracic endovascular aortic repair 3.5 months post index operation for residual descending aortic dissection with a maximum aortic diameter of 36 mm at zone 8. Thirty-eight months after her index repair, she presented to the ED with worsening chest pain. Electrocardiogram demonstrated inferior ST-segment elevation myocardial infarction. She died a week later after declining cardiac catheterization.

### Patient 7

A 65-year-old woman with a history of hypertension and hyperlipidemia presented with sudden-onset back pain, chest tightness, jaw pain, dyspnea, and nausea. She had never smoked and had no recent alcohol use. Vital signs were stable and no significant physical exam findings. Notably, her family history was positive for death from ruptured abdominal aortic aneurysm in father at age 89 and sudden cardiac death in paternal grandfather at age 54. No previous aortic imaging exists.

CTA demonstrated a Type A ascending aortic dissection with a diameter of 4.4 cm with extension through the arch, right innominate artery, and down to the right common and external iliac arteries. Genetic testing identified an MYLK W1352R VUS. The patient underwent ascending aorta, proximal arch repair, and aortic valve resuspension due to intraoperative transesophageal echocardiography demonstrating aortic insufficiency.

On POD4, the patient sustained multiple small embolic infarcts documented on magnetic resonance imaging, likely from circulatory arrest. The patient developed cognitive deficits including word-finding difficulty and slurred speech. Echocardiogram and CTA 6 months later showed stable cardiac function and aortic size.

### Patient 8

A 74-year-old woman with a history of hypertension, hyperlipidemia, hypothyroidism, and diabetes mellitus presented with a near-syncopal episode, shortness of breath, and throat tightness; she was initially evaluated for anaphylaxis and treated with epinephrine, diphenhydramine, methylprednisolone, and albuterol before a CTA ordered to rule out pulmonary embolism incidentally revealed an ascending aortic and root aneurysm (6.1 cm) with frank rupture at the distal ascending aorta, hemopericardium, and Type A intramural hematoma.

Genetic testing confirmed a pathogenic MYLK V1522A variant and a COL5A2 intronic VUS. Her mother died after heart surgery and her son (patient 4) has history of TAA with the same MYLK variant. Interestingly, patient 4's testing (through Yale Medical Genetics in 2021) noted the V1522A MYLK variant as VUS, whereas patient 8's testing (through Invitae in 2023) noted it as pathogenic.

The pathology was consistent with a chronic Type A dissection; imaging demonstrating inspissated clot within the greater curvature of the aorta and the original intimal tear having evolved into a walled-off pseudoaneurysm, consistent with a process at least several weeks old at the time of presentation. She was managed overnight with anti-impulse therapy (esmolol and nicardipine infusions) and diuresis for volume overload before elective operative scheduling the following morning.

The patient underwent emergent ascending aorta and hemiarch replacement via right axillary cannulation with DHCA (21 minutes). The aortic valve was resuspended. Postoperatively, she required quadruple vasopressor support, had transient anisocoria, and developed acute confusion and mild acute kidney injury. She was extubated on POD1 and discharged to a skilled nursing facility on day 7 without reoperation. She died at an unknown time after last telemedicine follow-up (71 months).

### Expert commentary

Patient 8 is the sole pathogenic MYLK carrier presenting at the oldest age (74 years) with what proved to be a chronic Type A dissection. She was discovered incidentally after an atypical near-syncopal episode, survived surgery, and died at an unknown date and undetermined cause. Her mother died following cardiac surgery, and her son (patient 4) has an identical MYLK variant and TAA, confirming vertical transmission and supporting cascade genetic screening of first-degree relatives. Across the entire series, 5 of 8 patients (63%) died during follow-up: 2 from cardiac causes, and 3 from undetermined causes. Notably, no death has been directly attributable to aortic disease, underscoring the methodological challenge of ascribing mortality in this complex population.

## Discussion

In our institutional registry of patients with aortic aneurysm with genetic testing from January 2012 to December 2023 (N = 1,048), MYLK carriers (n = 20) had nominally elevated rates of acute aortic syndrome compared with background rate (40% vs 18.7%; *P* = 0.037) and Type A dissection (30% vs 12%; *P* = 0.028); neither survived false discovery rate correction.

These 8 cases provide phenotypic context and reveal 4 patterns: 1) dissection without aneurysm, where 2 patients dissected at 40 mm, below any guideline threshold; 2) accelerated reoperation, with a median 7 months vs 24 months in patients with non-MYLK dissection (N = 27); 3) oligogenic amplification, where 2 patients carried co-occurring ACTA2 VUS; and 4) familial penetrance with intrapedigree reclassification, where patients 4 and 8 share MYLK V1522A, classified VUS by Yale in 2021 and pathogenic by Invitae in 2023. Together, these findings argue that MYLK VUS should not be interpreted as clinically reassuring but may warrant lower surveillance thresholds and cascade genetic screening.

**Table 1:** Case Series Patient Summary

Pt	Age, y	Sex	Variant	Type	Max Diam.	Dissection Extent	Family History	Primary Surgery	Reoperation	Outcome
1	29	M	MYLK p.R538W (VUS)	A	55 mm	Ascending + arch	Yes	Root + Asc + Hemiarch	No	Died (14 mo, cause unknown)
2	40	M	MYLK p.G1698R (VUS); ACTA2 p.I284N (VUS)	B	48 mm	Descending (limited)	ND	Descending replacement	Yes (14 mo)	Alive
3	41	M	MYLK p.G139T (VUS)	B	52 mm	Arch + descending to celiac	ND	Arch + Descending	Yes (7 mo)	Died (48 mo, cause unknown)
4	50	M	MYLK p.V1522A (VUS)^a^	A	40 mm	Root to bilateral iliac (extensive)	Yes	Asc + Hemiarch	No	Alive
5	59	M	MYLK p.C1339F (VUS); ACTA2 intron 2 (VUS)	A	40 mm	Root to pelvis (extensive)	ND	Asc + Hemiarch	No	Died (18 mo, CHF)
6	61	F	MYLK p.K241E (VUS)	A	44 mm	Root to infrarenal aorta + brachiocephalic occlusion	ND	Root + Asc + Hemiarch	Yes (3.5 mo)	Died (38 mo, STEMI/comfort care)
7	65	F	MYLK p.W1352R (VUS)	A	44 mm	Root to right iliac (extensive)	Yes	Ascending replacement	No	Alive
8	74	F	MYLK p.V1522A (Pathogenic)^a^; COL5A2 intronic (VUS)	A	61 mm	Ascending + arch only (IMH)	Yes	Asc + Hemiarch	No	Died (>71 mo, cause unknown)

Asc = ascending; CHF = congestive heart failure; IMH = intramural hematoma; Max Diam = maximum diameter; MYLK = myosin light chain kinase; ND = no disease; STEMI = ST-segment elevation myocardial infarction; VUS = variant of uncertain significance.

a Patient 4 and patient 8 are son and mother, respectively. Note that the MYLK variant was classified differently across their testing (Yale Medical Genetics in 2021 for patient 4, Invitae in 2023 for patient 8).

## Funding Support and Author Disclosures

The authors have reported that they have no relationships relevant to the contents of this paper to disclose.
